# Perinatal adversities as a common factor underlying the association between atypical laterality and neurodevelopmental disorders: A developmental perspective

**DOI:** 10.1111/psyp.14676

**Published:** 2024-08-28

**Authors:** Jad Hamaoui, Sebastian Ocklenburg, Hervé Segond

**Affiliations:** ^1^ Azrieli Research Center of Sainte‐Justine University Hospital Montreal Quebec Canada; ^2^ School of Psychoeducation University of Montreal Montreal Quebec Canada; ^3^ Department of Psychology MSH Medical School Hamburg Hamburg Germany; ^4^ ICAN Institute for Cognitive and Affective Neuroscience MSH Medical School Hamburg Hamburg Germany; ^5^ Institute of Cognitive Neuroscience, Biopsychology, Faculty of Psychology Ruhr University Bochum Bochum Germany; ^6^ Laboratoire de Psychologie des Cognitions, Department and faculty of Psychology University of Strasbourg Strasbourg France

**Keywords:** atypical laterality, developmental trajectories, hypothalamic–pituitary–adrenocortical axis, neurodevelopmental disorders, perinatal adversities, vestibular system

## Abstract

Several neurodevelopmental disorders are associated with a higher prevalence of atypical laterality (e.g., left‐handedness). Both genetic and non‐genetic factors play a role in this association, yet the underlying neurobiological mechanisms are largely unclear. Recent studies have found that stress, mediated by the hypothalamic–pituitary–adrenal (HPA) axis, could be linked to laterality development. These findings provide an opportunity to explore new theoretical perspectives on the association between atypical laterality and neurodevelopmental disorders. This article aims to provide a theoretical framework demonstrating how perinatal adversities could disrupt the typical developmental trajectories of both laterality and neurodevelopment, potentially impacting both the HPA axis and the vestibular system. Additionally, we argue that the relationship between atypical laterality and neurodevelopmental disorders cannot be understood by simply linking genetic and non‐genetic factors to a diagnosis, but the developmental trajectories must be considered. Based on these ideas, several perspectives for future research are proposed.

## Abstract 2

Atypical laterality (e.g., left‐handedness) is more prevalent in neurodevelopmental disorders. However, the nature of this relationship is unclear. This article aims to provide a comprehensive theoretical framework, adopting a developmental perspective, that demonstrates how perinatal adversities could explain the association between atypical laterality and neurodevelopmental disorders.

## 1 INTRODUCTION

Laterality reflects various asymmetries that can manifest at the behavioral level (e.g., handedness; McManus, 2019; Papadatou‐Pastou et al., 2020), the functional level (e.g., left hemisphere dominance for language; McManus, 2019), and the structural level (e.g., larger planum temporale in the left hemisphere; Kuo & Massoud, 2022). The ontogenetic mechanisms of laterality, the processes involved in its origin and development, are complex (Ocklenburg & Güntürkün, 2024). The different hypotheses of ontogenetic mechanisms of laterality can be summarized into two perspectives that are not mutually exclusive. The first view postulates that genetics and randomness are the major factors underlying functional and behavioral asymmetries in humans (McManus, 2021). The second perspective suggests that environmental factors such as prenatal environment, early sensorimotor experiences, and sociocultural factors play an important role in the development of laterality (Michel, 2021).

Over the past years, the interest in studying laterality has increased considerably, driven by several findings that reveal a higher prevalence of non‐right handedness (i.e., left‐ and mixed‐handedness) among individuals with neurodevelopmental disorders. Atypical handedness (i.e., non‐right handedness) has been associated with neurodevelopmental disorders such as developmental dyslexia (DD, for meta‐analyses see Abbondanza et al., 2023; Eglinton & Annett, 1994; Packheiser et al., 2023), developmental coordination disorder (DCD, see for meta‐analysis Darvik et al., 2018), intellectual disability (see for meta‐analysis Papadatou‐Pastou & Tomprou, 2015), and autism spectrum disorder (see for meta‐analysis Markou et al., 2017). Furthermore, there is also evidence of atypical functional and structural lateralization among individuals with DD (Berretz, Wolf, et al., 2020; Bishop, 2013; Mundorf et al., 2021) and DCD (Biotteau et al., 2016; Hodgson & Hudson, 2017), among other disorders. An increase in atypical laterality can also be observed in some psychiatric disorders, such as schizophrenia and schizotypal personality (see for meta‐analyses Hirnstein & Hugdahl, 2014; Somers et al., 2009).

**FIGURE 1:**
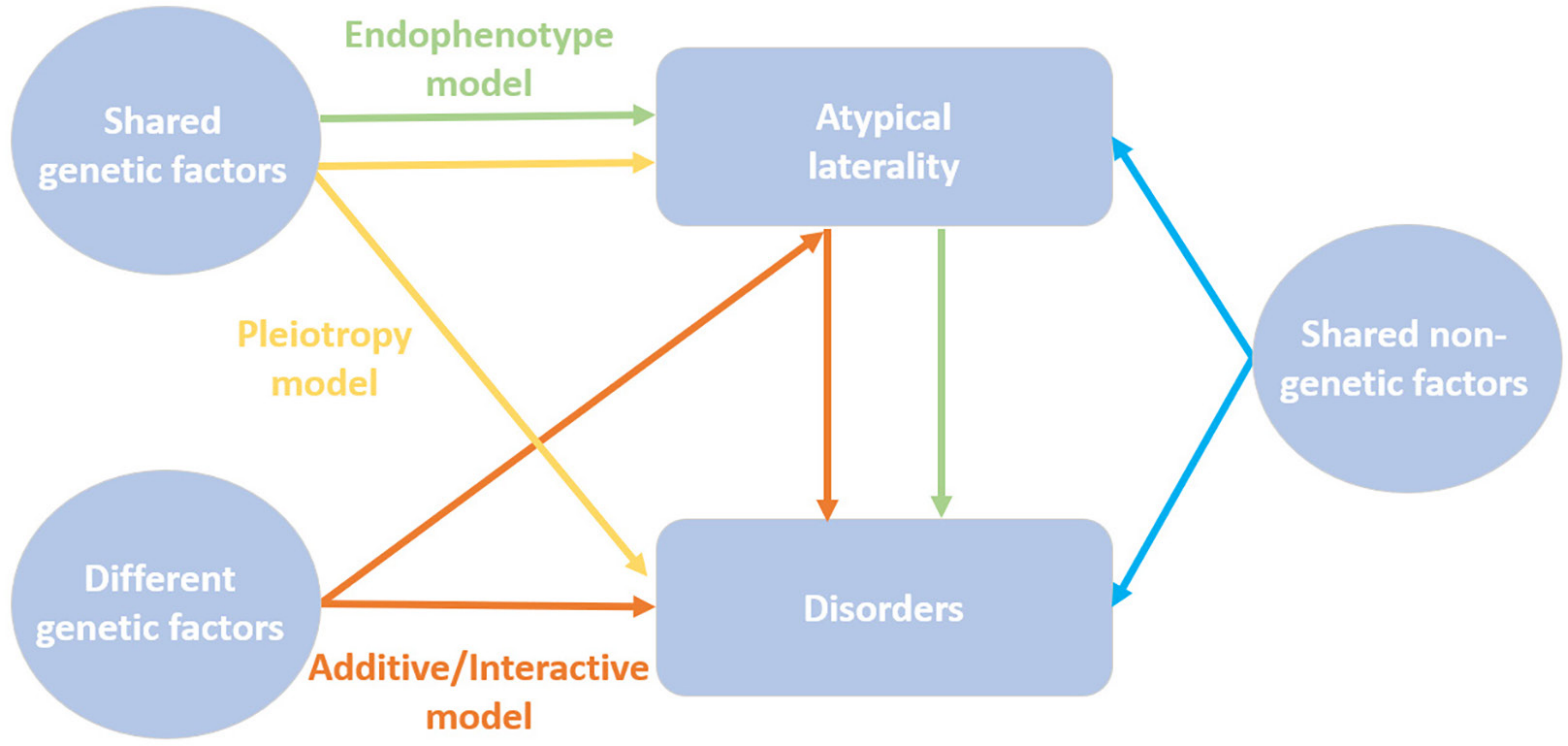
Different associations between atypical laterality and disorders. The endophenotype model posits that genetic factors lead to atypical laterality, subsequently influencing the development of various disorders. The pleiotropy model suggests that shared genetic factors contribute to both atypical laterality and disorders, without establishing a direct causal link between these phenotypes. Meanwhile, the additive/interactive model posits that genetic factors influencing disorders operate independently of those impacting atypical laterality, but the latter is considered an independent and additive risk factor contributing to the development of disorders. In addition to the shared and non‐shared genetic influences, it is considered that atypical laterality and disorders could also share common non‐genetic factors.

Considering the assumption that there is a genuine relationship between atypical laterality and disorders (i.e., neurodevelopmental and psychiatric disorders), there is no clear consensus on how they are related (Mundorf & Ocklenburg, 2021, p. 3; Ocklenburg et al., 2024). Bishop (2013) proposed different theoretical models to explain the possible relationship between neurodevelopmental disorders and atypical laterality, more precisely between language impairments and weak lateralization. There may be a causal relation, where weak cerebral lateralization exerts a direct influence on language impairments. Cerebral lateralization and language impairments may share the same genes (i.e., the endophenotype model) or not (i.e., the additive/interactive risks model). Another perspective suggests that language impairments, which have a genetic basis, favor the development of atypical weak lateralization (i.e., the neuroplasticity model). Lastly, it could also be posited that language impairments and atypical lateralization are not linked, but share the same origin, which is likely to be genetic (i.e., the pleiotropy model). The current evidence supports to some extent a combination of the endophenotype, additive/interactive, and pleiotropy models (Ocklenburg & Mundorf, 2023; see Figure 1). It seems unlikely that the neuroplasticity model is correct, given that a disorder would have to appear before the emergence of atypical laterality. However, this is not supported by the literature (e.g., Crow et al., 1996; Irani et al., 2023; Lindell, 2020). Recent findings, however, indicate that there is some overlap between the genetic factors associated with handedness and disorders, alongside independent genetic factors (Cuellar‐Partida et al., 2021). While there is evidence showing shared genetic factors associated with atypical laterality and disorders such as DD, autism spectrum disorder, and schizophrenia (Berretz, Wolf, et al., 2020, p. 221; Sha, Schijven, Carrion‐Castillo, et al., 2021; Sha, Schijven, & Francks, 2021; Wiberg et al., 2019), it is noteworthy that genetic influences cannot completely explain the atypical lateralization found among several of these disorders (Berretz, Wolf, et al., 2020; Cuellar‐Partida et al., 2021; Kong et al., 2020). This has led some researchers to explore additional factors beyond genetics and to examine non‐genetic influences in the complex relationship between atypical laterality and disorders (Mundorf & Ocklenburg, 2021, p. 107; see Figure 1). Within these non‐genetic influences, perinatal adversities emerge as a compelling candidate warranting exploration (Berretz, Wolf, et al., 2020; Bishop, 2013; Schmitz et al., 2017).

## 2 PERINATAL ADVERSITIES, ATYPICAL LATERALITY, AND NEURODEVELOPMENTAL DISORDERS

### 2.1 Past theoretical frameworks and recent evidence

Several influential theories from the 1970s suggested that non‐right‐handedness could be a consequence of pathological events occurring between the prenatal period and birth, which are usually regrouped under the pregnancy complications and birth stressors (PCBS) label. The first proposed theory was called: “Early brain insult,” where non‐right‐handedness is considered a result of neurological impairment during pregnancy (Bakan, 1971, 1977; Bakan et al., 1973). The higher occurrence of non‐right‐handedness is indirectly explained by a larger vulnerability of the left over the right hemisphere. The former is supposed to have a greater need for oxygen due to a more active metabolism (Bakan et al., 1973). Also, the late development of the left hemisphere over the right suggests it is more vulnerable to external influences (e.g., Bisiacchi & Cainelli, 2021). Such influences, like hypoxia (i.e., oxygen deprivation), could lead to non‐right‐handedness. Because handedness is associated with motor lateralization in early neonatal life (Bisiacchi & Cainelli, 2021), minor PCBS coupled with hypoxia could lead to a unilateral brain insult localized in the left hemisphere. This may then alter the functioning of the contralateral hand resulting in a shift from right‐handedness to left‐handedness (Bakan, 1971; Bakan et al., 1973; Satz, 1973). Since a co‐occurrence has been observed between hypoxia and disorders such as autism spectrum disorder, intellectual disability, and schizophrenia (e.g., Giannopoulou et al., 2018), these observations have paved the way for hypotheses linking PCBS, atypical handedness, and disorders. It should be noted that Bakan suggested that all left‐handers suffered from early brain damage. This can be considered an extreme hypothesis and likely incorrect since, on average, left‐handers do not show cognitive deficits or motor impairment related to early brain insult (Bishop, 1990a, p. 90–92; for further discussion see McManus, 1983).

Satz (1972, 1973) diverges from Bakan by proposing the “pathological left‐handedness” theory. It can be considered less extreme as it does not postulate that all left‐handers suffer from PCBS. Based on the hypothesis that brain damage may determine the development of handedness in some individuals, Satz stipulated that there would be two types of pathological handedness: the pathological left‐handers and the pathological right‐handers. While the former reflects left‐brain damage among natural right‐handers, the latter consists of natural left‐handers with right‐brain damage (Satz, 1973). Contrary to Bakan's theory, there is no need to consider the left hemisphere as more vulnerable to brain insults to explain the higher prevalence of pathological left‐handedness over right‐handedness. Given that right‐handers outnumber left‐handers by approximately ninefold (Papadatou‐Pastou et al., 2020), this disparity could account for the higher prevalence of left‐handedness due to a brain injury. However, while these two possible pathways propose an underlying mechanism for atypical handedness, they cannot explain the higher prevalence of non‐right‐handedness among individuals with neurodevelopmental and psychiatric disorders. This is due to the fact that these disorders are not necessarily related to unilateral or bilateral brain insult (Previc, 1996). It is noteworthy that Batheja and McManus (1985) offered an alternative mechanism to the pathological left‐handedness theory. They suggested that early brain insults increase biological noise. This thus leads to a higher fluctuating asymmetry, which in turn increases the likelihood of chance playing a role in the development of handedness (Batheja & McManus, 1985; Previc, 1996).

Vargha‐Khadem et al. (1985) found that all patients with prenatal and early postnatal left‐hemisphere lesions developed left‐handedness, regardless of the lesion's severity. These data support the concept of “pathological left‐handedness” (for similar results see Orsini & Satz, 1986). More recently, an excess of mixed‐handedness and left‐handedness was found among premature children with brain injuries compared to those with no injuries (Marlow et al., 2019). These findings could also support Batheja and McManus' (1985) explanation that early brain damage may increase the implication of randomness in the development of handedness. However, another recent study failed to find an association between brain injury and atypical handedness (van Heerwaarde et al., 2020). The authors suggest this may be due to low statistical power, however, it is possible that these results indicate that atypical handedness has different origins, resulting from genetic and environmental pathological factors (van Heerwaarde et al., 2020).

Other early complications and stressors such as low gestational age (i.e., prematurity), low birth weight, and deteriorated neonatal health may disrupt a typical developmental trajectory of the fetus and the neonate, are also considered to be risk factors for neurodevelopmental disorders (Cha et al., 2022; Modabbernia et al., 2016). This could suggest that PCBS can lead to an atypical development of laterality. Several findings showed that gestational age and birthweight are related to handedness (see for meta‐analyses Domellöf et al., 2011; Searleman et al., 1989). A higher prevalence of non‐right‐handedness is found among premature children (Burnett et al., 2018; Marlow et al., 2019; van Heerwaarde et al., 2020) and children with low birth weight (James & Orlebeke, 2002; O'Callaghan et al., 1987, 1993; Powls et al., 1996). Furthermore, deteriorated neonatal health reflected by a low Apgar score is shown to increase the prevalence of left‐handedness (Dragović et al., 2013; Schwartz, 1988). Other studies failed, however, to find an association between PCBS and handedness (Annett & Ockwell, 1980; Dellatolas et al., 1991; Ehrlichman et al., 1982; Levander et al., 1989; McManus, 1981; Tan & Nettleton, 1980; Van Der Elst et al., 2011). Indeed, these studies, using various study designs (e.g., surveys and cohort studies), and examined a range of perinatal complications (e.g., prematurity, low birthweight, birth order, older maternal age, and hypoxia) and outcomes (e.g., hand preference and eye preference), found no significant association between perinatal adversities and atypical laterality. For instance, Nicholls et al. (2012) investigated the relationship between birth stress (e.g., low birth weight and any behavioral signs of cerebral damage), hand preference, and cognitive ability (e.g., verbal and nonverbal skills). They analyzed data from the British Cohort Study with a sample size of approximately 10,000 children and found that while birth stress affected cognitive ability, it did not influence hand preference.

These conflicting results may be in part due to the different methodologies applied for assessing handedness and PCBS (Coren et al., 1982; Elliott, 1992; Levander et al., 1989; Marcori & Okazaki, 2020; Searleman et al., 1989). Firstly, some studies used non‐validated handedness measurements such as parental reporting, or the sole assessment of the writing hand. Secondly, PCBS were either self‐reported or indicated by the parents, which are both less accurate than hospital records. Thirdly, several studies grouped all forms of PCBS under a single category. This approach is not ideal since each PCBS may uniquely affect handedness. Fourthly, some studies conducted their investigation on small sample sizes, resulting in low statistical power. In addition to these methodological limitations, we can offer two theoretical ones. The first is that most studies only compared the prevalence of right‐ versus left‐handedness or right‐ versus non‐right handedness. However, this dichotomized classification, especially grouping left‐ and mixed‐handedness, must be treated with caution. Some authors suggest that PCBS might not lead to a complete shift from right‐ to left‐handedness. Instead, PCBS might only reduce the bias toward the right‐hand preference, leading to mixed‐handedness (Coren et al., 1982; Domellöf et al., 2011; Hicks et al., 1980; Searleman et al., 1989). On top of that, it has been shown that among extremely preterm children (<28 weeks) assessed at 10 years of age, mixed‐handed individuals were associated with an increased risk of cognitive and motor difficulties (Burnett et al., 2018). These results suggest that mixed‐handed children who experienced PCBS may have different developmental trajectories than their left‐ and right‐handed peers. As a result, mixed‐handedness should be assessed on its own (Domellöf et al., 2011; Marcori & Okazaki, 2020; Van Der Elst et al., 2011). The second theoretical limitation is the prevailing measurement of only one dimension of handedness, namely hand preference, neglecting hand performance. Measuring the latter along a continuum may be a more sensitive approach when studying the influence of PCBS (Bishop, 1984). It is suggested that PCBS may reduce right‐hand ease of use, but not necessarily cause a hand preference switch from right to left (Domellöf et al., 2011; Ross et al., 1992; Van Der Elst et al., 2011).

While taking into consideration the above limitations, some studies investigated the implication of prematurity, birth weight, and neonatal health as assessed with the Apgar test, on both handedness (i.e., hand preference and hand performance) and neurodevelopmental disorders, specifically DD and DCD (Hamaoui, 2022, p. 147; Hamaoui et al., 2022). The authors found that very low gestational age (<32 weeks gestation) and very low birth weight (<1500 g) were associated with a left‐hand preference and DCD, but not DD. These results support previous studies that reported a higher rate of atypical handedness and motor impairments among individuals with PCBS (de Kovel et al., 2019; Domellöf et al., 2011; Dragović et al., 2013; Zwicker et al., 2013). Nonetheless, very poor neonatal health (Apgar test score <4) assessed at 5 min, but not at 1 min, was only related to a left‐hand preference and a weak hand performance (Hamaoui, 2022, p. 148–149; for similar results on the association between atypical handedness and the Apgar test, see Dragović et al., 2013; Schwartz, 1988).

It appears clear that PCBS are related to atypical handedness, and the aforementioned results suggest that some PCBS (i.e., very low gestational age and very low birthweight) could be common factors associated with both atypical laterality and neurodevelopmental disorders.

### 2.2 Novel theoretical hypotheses and neurobiological mechanisms

This section proposes several theoretical hypotheses, based on recent evidence, which are not necessarily mutually exclusive, to describe potential connections between PCBS, atypical laterality, and neurodevelopmental disorders. Some authors suggest that stress, which is regulated by the hypothalamic–pituitary–adrenal (HPA) axis, could be a contributing factor to the association between atypical laterality and neurodevelopmental disorders. Ocklenburg et al. (2016) proposed two non‐genetic models showing how stress could modulate cerebral lateralization, and how it could explain the association between atypical laterality and disorders. In the first, the hormonal model, cortisol influences the interhemispheric connection, which may reduce cerebral lateralization. In the second, the cognitive emotionality model, stressful situations are associated with a greater right hemisphere response, via an interaction between stress and emotional lateralization. In the same vein, Berretz, Wolf, et al. (2020) considered that alterations in the HPA axis might lead to the manifestation of atypical cerebral lateralization. The mechanism underlying this theoretical model is a disturbance in the typical levels of cortisol in the organism. It is stipulated that early life stress and chronic stress are risk factors for a reduction of cerebral lateralization, as well as for neurodevelopmental and psychiatric disorders. The relationship between stress, cortisol, and cerebral lateralization appears to be complex, but recent studies provide evidence that acute and chronic stress can influence to some extent hemispheric asymmetries on a behavioral and neural level (Berretz et al., 2022a, 2022b; Berretz, Packheiser, et al., 2020; Mundorf et al., 2020, 2023; Mundorf & Ocklenburg, 2023). It is noteworthy that stress has been linked to the development of laterality in various non‐human species, including non‐human primates, non‐primate mammals, birds, and fishes (for review, see Ocklenburg et al., 2016).

Interestingly, elevated levels of cortisol predict delayed fetal growth, fetal activity, and importantly low gestational age and low birthweight (Field et al., 2006; Field & Diego, 2008). Cortisol can directly cross the placenta and therefore mothers' prenatal cortisol levels are associated with their fetus' cortisol levels, and consequently with their newborns' levels (Field & Diego, 2008). It was found that perinatal adversities lead to a permanent modification of the HPA axis (Kapoor et al., 2006). Very preterm school‐age children were found to have an altered HPA axis functioning (Brummelte et al., 2015). The right hemisphere is linked to the HPA axis, and cortisol secretion is mainly under the excitatory control of this hemisphere (Hecht, 2010). For example, when exposed to stressful stimuli, 8–9‐year‐old children with low birth weight show greater blood flow to the right hemisphere than to the left one (Jones et al., 2011). Mundorf et al. (2020) found that in rats, early‐life stress‐induced atypical asymmetries in turning behaviors favoring the left side. This suggests that stress exposure leads to greater activation of the right hemisphere. In line with these results, maternal prenatal stress is found to be positively related to fetal left‐handed self‐touch (Reissland et al., 2015). Furthermore, it is well known that PCBS disrupt motor and language development and increase the risk of neurodevelopmental disorders (Cha et al., 2022; Modabbernia et al., 2016; Wallois et al., 2020), which are generally associated with the dysregulation of the HPA axis (Cartier et al., 2016; Theodoridou et al., 2021). Therefore, it can be stipulated that PCBS, via the disruption of both the HPA axis (Kapoor et al., 2006) and typical neurodevelopment (Wallois et al., 2020), can lead to atypical laterality and neurodevelopmental disorders.

**FIGURE 2:**
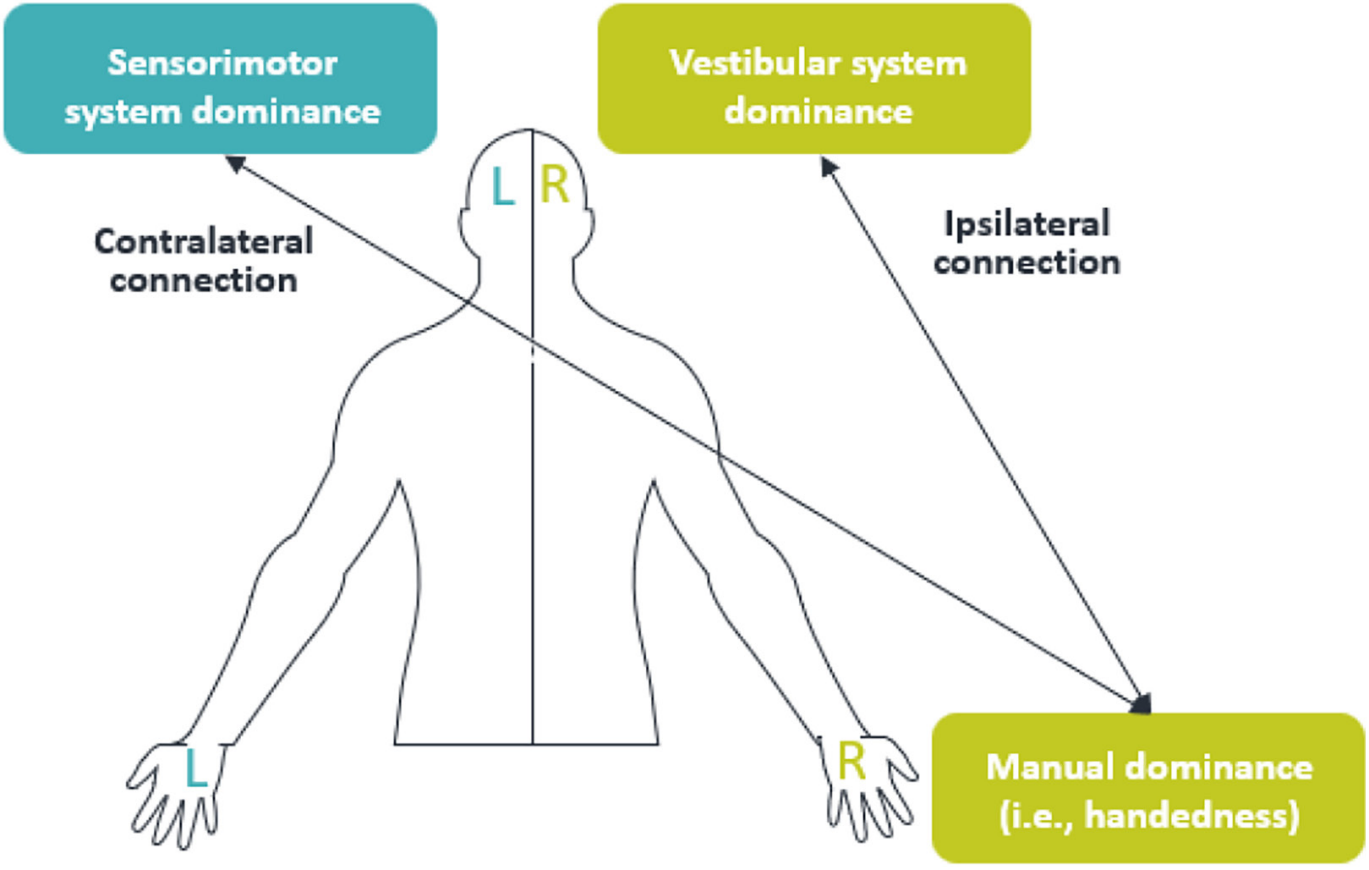
Vestibular and sensorimotor system dominance in relation to manual dominance in right‐handers. L, left; R, right.

The vestibular system, which is located in the inner ear, is involved in the sensory inputs for balance control and cognitive functions that include spatial memory, orientation, and navigation (Brandt & Dieterich, 2015). Newborns aged from 1 to 5 days show a Moro reflex asymmetry, where the right arm starts to move before the left (Rönnqvist, 1995). These results suggest that there is an early spinal asymmetry related to the vestibulospinal system. Since the Moro reflex is connected to the vestibular system, it can be assumed that vestibular lateralization during gestation leads to newborn movement and posture asymmetries (Rönnqvist, 1995). Furthermore, on a functional level, some evidence shows that the vestibular and the motor lateralization are related. Among adults, using caloric irrigation in positron emission tomography (Dieterich et al., 2003) and auditory evoked vestibular otolith stimulation in fMRI (Janzen et al., 2008), it was found that there is a right‐hemisphere dominance for the vestibular system in right‐handers, and a left hemisphere dominance in left‐handers (for a meta‐analysis see Zu Eulenburg et al., 2012). Thus, the vestibular dominance seems to be ipsilateral to handedness, and therefore contralateral to the sensorimotor hemispherical dominance (i.e., the left hemisphere controls the right hand and vice versa for the left hand; see Figure 2). These findings suggest a possible relation between vestibular and motor lateralization.

Since the vestibular system matures early during gestation, before the development of the hand preference, Brandt and Dieterich (2015) proposed a hypothesis that early vestibular system lateralization determines later sensorimotor lateralization. The authors suggested that each of the higher vestibular functions (e.g., spatial memory, orientation, and navigation) and handedness require their own coordinate system. The former is allocentric and responsible for self‐localization in the environment, whereas the latter is egocentric and responsible for object manipulation. Their functional lateralization in opposite hemispheres enables them to operate independently from one another, allowing for optimization of both functions during development (Dieterich & Brandt, 2018a). Thus, an early vestibular lateralization will lead to a well‐established perceptive and motor functional asymmetry (Brandt & Dieterich, 2015; Dieterich et al., 2003).

**FIGURE 3:**
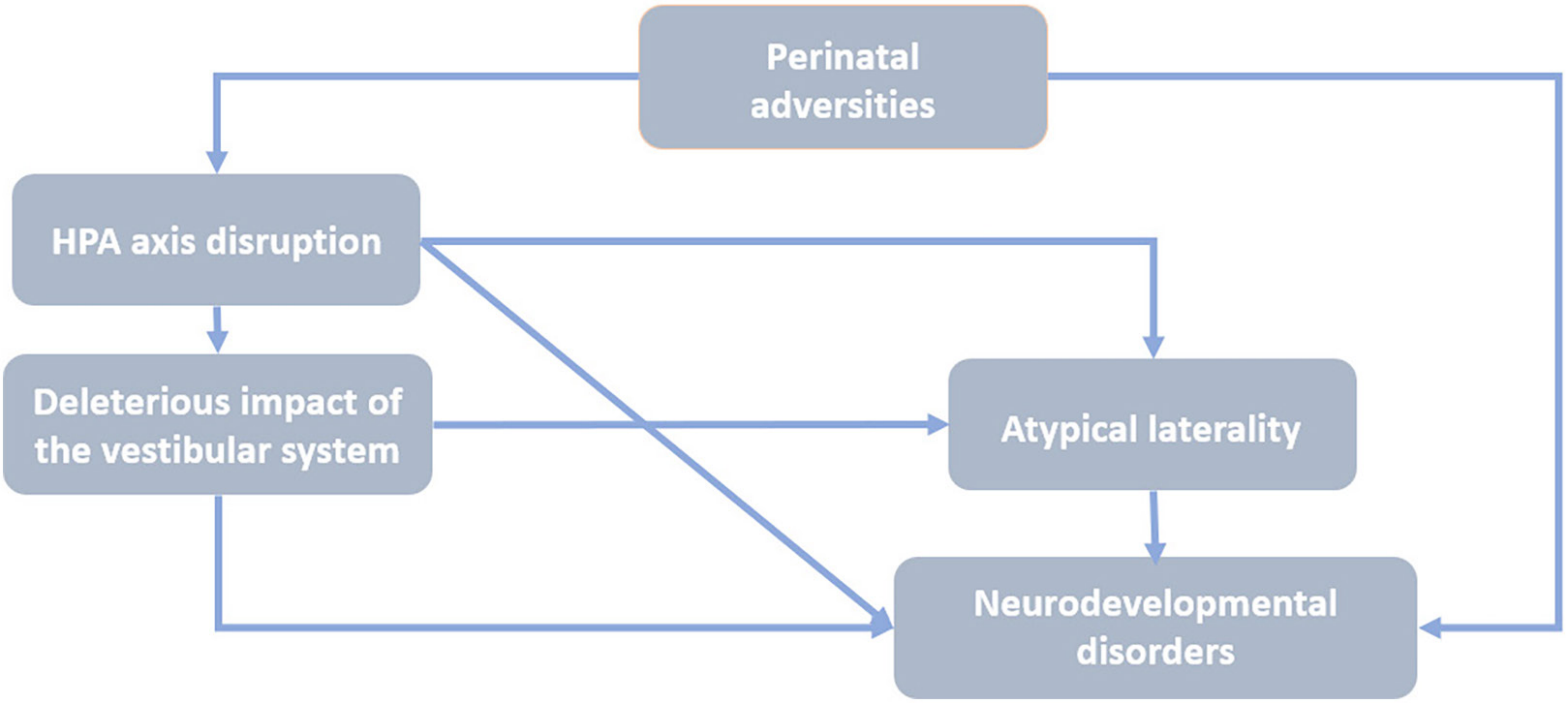
The influence of perinatal adversities on the HPA axis and the vestibular system, and subsequently on atypical laterality and neurodevelopmental disorders. Stress is mediated by the hypothalamic–pituitary–adrenal (HPA) axis, with elevated cortisol levels being associated with perinatal adversities. These adversities can permanently modify the functioning of the HPA axis. Moreover, the HPA axis is interconnected with the vestibular system, and excessive or inappropriate stress can have deleterious effects. Additionally, both the HPA axis and vestibular system are observed among individuals with neurodevelopment disorders and hypothesized to be linked to the development of laterality. This implies that dysfunctions within these systems could lead to atypical laterality. Finally, perinatal adversities disrupt language and motor development and are associated with neurodevelopmental disorders.

This hypothesized relationship between the vestibular and motor lateralization could explain how PCBS are connected to atypical handedness. Activation of the HPA axis has been shown to be connected to the vestibular system, where excessive or inappropriate stress can have a deleterious impact (Saman et al., 2020). Therefore, prenatal and perinatal stress events could disturb vestibular lateralization. This hypothesis is supported by the evidence that premature children are found to exhibit a weaker visuospatial attentional bias compared to their peers, reflecting an atypical functional lateralization of the right hemisphere (Davis et al., 2022). Notably, visuospatial attention and vestibular processing are partly related to each other and are both lateralized in the right hemisphere (Karnath & Dieterich, 2006). Thus, PCBS may disrupt early vestibular lateralization, which could alter the hemispherical asymmetries of other functions. Additional findings also support the link between atypical vestibular lateralization and other atypical asymmetries. It was shown that atypical functional lateralization is found among individuals with DD characterized by visuospatial deficits, spatial dysgraphia, dyscalculia, and finger agnosia but without dysfunctional language (Pirozzolo, 1979, as cited in Bemporad & Kinsbourne, 1983). Furthermore, visual motion sensitivity, which is related to the vestibular system (Dieterich & Brandt, 2018b), is found to be more impaired in non‐right‐handers with DD compared to the control group (Richardson, 1995). Therefore, as mentioned by Previc (1991), atypical cerebral lateralization and non‐right handedness are likely to be present only in specific subtypes of disorders associated with atypical vestibular lateralization, for example, the visuospatial deficit subtype of DD. Furthermore, vestibular cortical areas are linked to the motor and pre‐motor cortex used for balance and voluntary movement coordination (Carmona et al., 2009). PCBS are also shown to impact the maturation of the motor system (Wallois et al., 2020). It is possible, therefore, to explain how PCBS can be related to both atypical functional lateralization via vestibular lateralization and motor impairments via the alteration of motor and vestibular development. Figure 3 summarizes the novel theoretical hypotheses presented in this section regarding the neurobiological mechanisms that could explain the relationship between perinatal adversities, atypical laterality, and neurodevelopmental disorders.

### 2.3 Beyond genes and environment: Taking the developmental perspective

One may inquire into the extent to which each of the genetic and environmental factors could be implicated in the developmental trajectories of laterality. Genetic factors reflect the genetic variation among individuals in a population, whereas non‐genetic factors include environmental variations such as maternal effects, ontogenetic variation, and randomness (Graham, 2021). In their meta‐analysis, Searleman et al. (1989) found that birth stressors and handedness are very weakly associated and accounted for less than 1% of the variances. Similar results were once again found by de Kovel et al. (2019) who studied the role of early life factors on handedness in a very large sample (*n* = 500,000 approximately). The influence of intrauterine environment seems to be genuine but appears to be very small (de Kovel et al., 2019). McManus (2021) suggests that environmental factors could explain, at best, 1%–2% of the variance in handedness, while genetic factors are most likely to be the strongest contributor (McManus, 2009, 2021). However, genetic factors are limited in explaining most of the variances in the population, and the heritability of handedness is around 24% (Medland et al., 2009; Schmitz et al., 2022). Therefore, most of the variance in handedness is unaccounted for. Understanding what accounts for this unexplained variance is essential for gaining a clearer insight into the link between atypical laterality and neurodevelopmental disorders.

McManus (2002, 2021, 2022) emphasizes the role of randomness (i.e., fluctuating asymmetry) in the development of handedness. In genetics, most of the environmental component of the total phenotypic variance can be attributed to random developmental and stochastic variations, which involve the random behavior of molecules and cells in the developing organism (Graham, 2021). This can explain the differences between genetically identical individuals raised in the same environment. Developmental noise and randomness are also suggested to play a major role in the ontogenesis of handedness (McManus, 2022). Therefore, the nonlinear developmental processes of handedness could be partly deterministic but modified by true random variation (Graham, 2021). Stochastic variations in language lateralization are also discussed in Bishop and Bates (2020). Mitchell (2018) provides an overview of the influence of randomness on handedness, but also on other phenotypes like intelligence, personality, and neurodevelopmental disorders.

While randomness may account for some unexplained variance, additional factors, which are not easily identified by population and genetics studies, play an important role in the development of laterality. Population studies, though valuable, often oversimplify the physiological processes and their reciprocal interactions with the environment, which are crucial for understanding development (Michel, 2021). Moreover, such studies may not provide a comprehensive understanding of the underlying mechanisms. Considering the dynamic and probabilistic nature of development (Lewkowicz, 2011), detailed investigations of developmental stages are essential (Michel et al., 2018). Michel (2021) postulated that laterality is likely to emerge from the trajectories and dynamics of neural activity interacting with individual experiences, with genetics playing a very limited role in its initial development. The author suggested that the early and typical development of handedness is a consequence of a complex cascade of events involving prenatal and postnatal postural asymmetries, which contribute to the growth of early sensorimotor asymmetries of the arm and hand. Consequently, these early asymmetries will favor the use of a preferred hand for simple unimanual and later complex bimanual motor activities. These developmental cascades are suggested to start with the fetal posture, seen as one of the first plausible causes of handedness. After the 16th week of gestation, factors such as gravity and the uterus's shape restrict the fetus's position and movement (Ververs et al., 1994). Due to this restriction, the fetus adopts a cephalic (i.e., vertex) presentation, where the head is down and fixed in the mother's pelvis. While adopting this presentation, most fetuses turn their back to the left (i.e., left occiput uterine position), which will constrain the left arm movement and the leftward head turns (Previc, 1991). In contrast, fetuses that turn their back to the right (i.e., right occiput uterine position) will have their right arm movement constrained and the head turns oriented to the left. An asymmetrical fetal presentation will likely lead to lateral asymmetries in the organization of spinal synergies, which are related to the functional coordination between different muscles that produce a specific movement.

**FIGURE 4:**
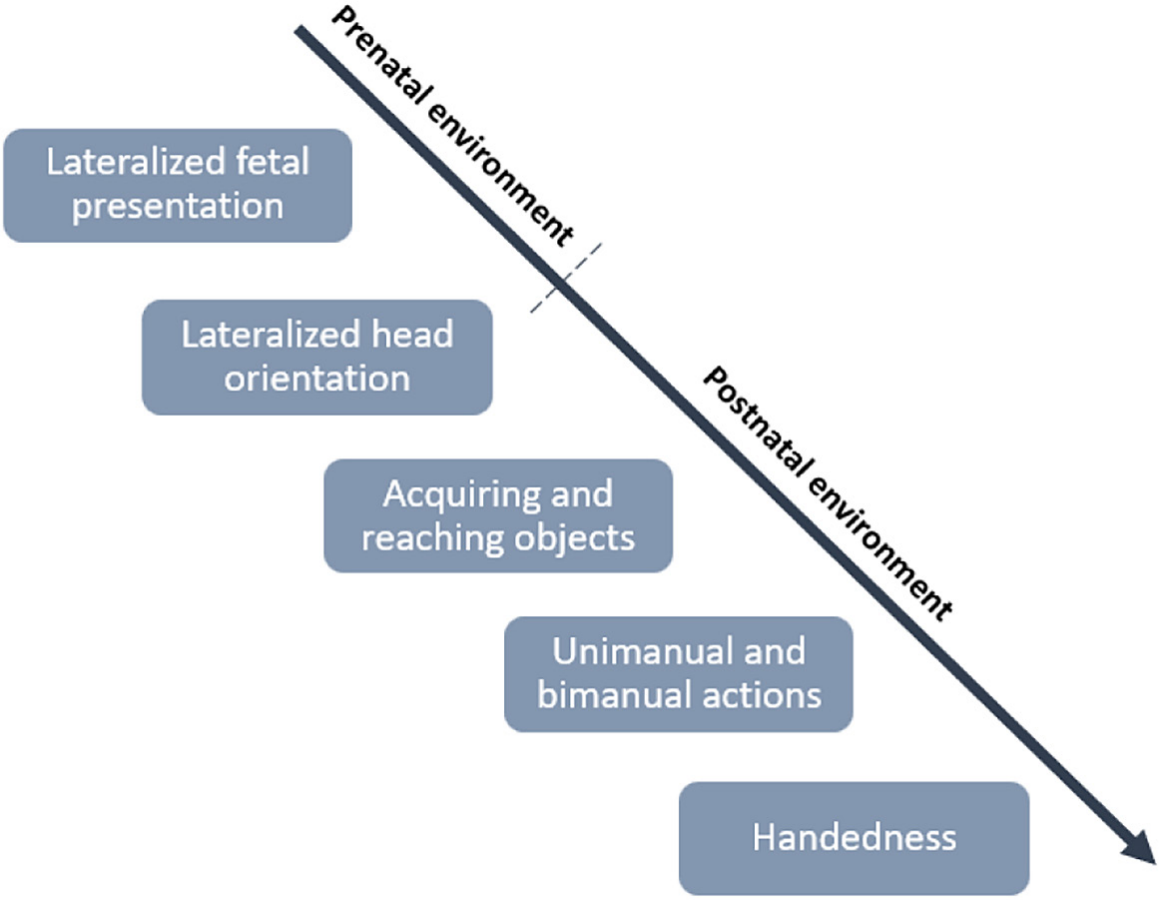
The cascade theory of handedness as suggested by Michel (2021). This figure shows the developmental cascades that lead to handedness. A lateralized fetal presentation will result in a head orientation preference. Since most fetuses adopt a leftward cephalic presentation, it will lead to prenatal and postnatal rightward head orientation bias. Afterward, this head orientation preference will lead to a right‐hand use for acquiring and reaching objects. Throughout the first years after birth, motor actions using the right hand will become more complex with the appearance of unimanual and then bimanual actions. The accumulation of sensorimotor experiences with the right hand will ultimately lead to the development of right‐handedness.

These asymmetries are likely to produce a directional prenatal and postnatal head‐turn (Rönnqvist et al., 1998). Newborn infants with a leftward cephalic presentation will exhibit a rightward head orientation preference in the supine position after birth, whereas newborns with a rightward cephalic presentation will exhibit a leftward head orientation preference in the postnatal supine position (Michel & Goodwin, 1979). A rightward head orientation preference is associated with right‐handedness, whereas a leftward head orientation is related to left‐handedness (Goodwin & Michel, 1981; Michel & Harkins, 1986). It is stipulated that the asymmetrical proprioceptive and visual experience of the hand are the mechanisms underlying the association between early head orientation preference and later handedness (Michel, 2021; Ocklenburg et al., 2010). During the first year of life, the hand that was in the visual field during the early head orientation preference will be preferred by infants for reaching and afterward for acquiring objects (Michel, Nelson, et al., 2013). Ultimately, these asymmetrical experiences will contribute to the development of handedness (Michel, 2021; Michel, Babik, et al., 2013; see Figure 4). Therefore, according to this view, the early development of handedness results primarily from interactions between the individual and their environment.

Notable, asymmetries caused by fetal presentation are the main contributors to the development of postnatal functional and behavioral lateralization, in this theory, rather than an already established cerebral lateralization (Michel, 2021). This theoretical approach is related to the Embodied cognition theory (for a brief review of Embodied cognition and handedness see Willems et al., 2014), where it is suggested that the early asymmetric sensorimotor activities during the development of handedness will lead to hemispheric variations in the neurophysiology of cognitive and emotional functions (Michel, Nelson, et al., 2013). On a side note, early environmental influences are also evident in studies on nonhuman species, such as research on chicks that has shown that light exposure during incubation can affect the development of visual laterality (see for review Güntürkün & Ocklenburg, 2017).

## 3 INTEGRATION OF FINDINGS: A DEVELOPMENT‐CENTRIC FRAMEWORK FOR CLINICAL LATERALITY RESEARCH

Having reviewed several key theoretical concepts and mechanisms presented in both past and recent literature, we will illustrate in this section their integration into a unified theoretical framework applicable to clinical research. Various factors have been identified as influential in the development of both laterality and neurodevelopmental disorders. These encompass genetic elements, environmental factors such as prenatal and perinatal adversities, and inherent randomness. Additionally, exploring the mechanisms through which perinatal adversities influence laterality and neurodevelopmental disorders suggests a potential connection between the HPA axis and the vestibular system.

Nonetheless, it is crucial to note that the impact of all of these factors is not straightforward; rather, it operates through intricate developmental cascades. Thus, understanding laterality requires a comprehensive approach that goes beyond just genes, environment, and randomness. While genetics and randomness constrain the developmental trajectories of handedness, individual‐environment coaction (e.g., early sensorimotor experience and sociocultural influences) affects both the direction and strength of handedness (Michel, 2021). In the context of atypical development, as observed in neurodevelopmental disorders, early events can disrupt subsequent development through cascading effects, wherein deficits in one domain, such as motor development, can lead to deficits in another, such as language development (Paterson et al., 2016). Consequently, we can suppose that the different developmental trajectories of laterality, too, will be shaped by early adverse events, through cascading events.

**FIGURE 5:**
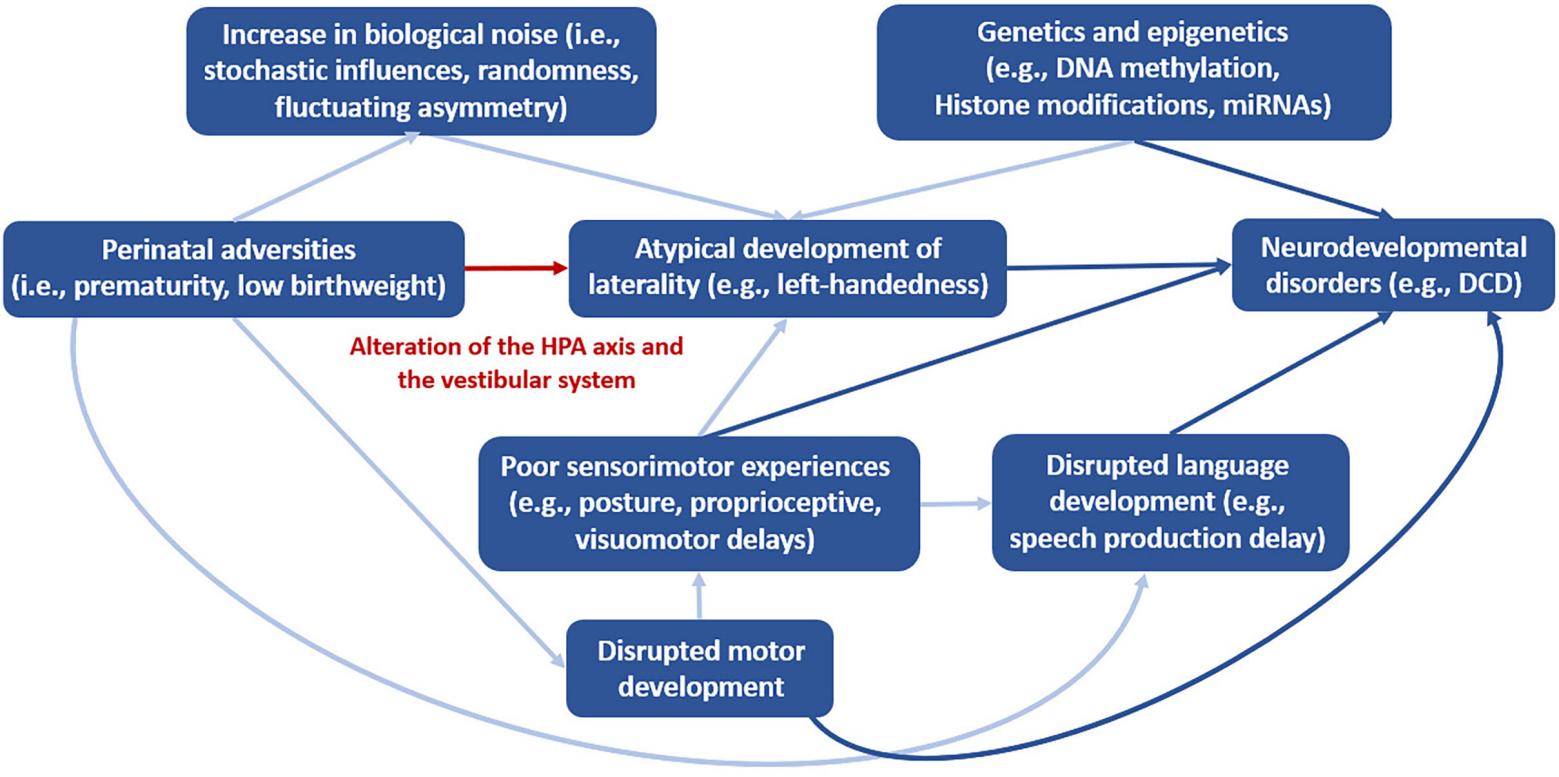
Theoretical framework presenting comprehensively the association between perinatal adversities, atypical laterality, and neurodevelopmental disorders. In this model, the relationships between the variables are primarily hypothetical and require validation through empirical research. Future investigation is needed to gain a deeper understanding of the specific developmental relationships among the various components of this model. For a presentation on how to test some of these proposed relationships using longitudinal studies and the appropriate assessments, refer to Michel (2018).

We present here a theoretical framework offering a comprehensive integration of the intricate interplay among genetic, environmental, and stochastic factors. This framework emphasizes the dynamic developmental processes that influence laterality, ultimately contributing to neurodevelopmental disorders (see Figure 5).

Similarly to Satz (1972, 1973), we find it reasonable to suggest both a non‐pathological and a pathological origin for atypical laterality (e.g., handedness; Schaafsma et al., 2009). For the former, it is likely that it is determined by rare intrinsic factors (e.g., genetics, Mcmanus et al., 2013) and environmental influences (Michel, 2021) favoring the motoric left side. For the latter, perinatal adversities could play a significant role by disrupting early fetal growth, leading to atypical development of laterality, motor, and language systems (Davis et al., 2022; Domellöf et al., 2011; Wallois et al., 2020; see Figure 5). The developmental cascade events suggested by Michel (2021) could be impacted by the PCBS. On a behavioral level, Moro reflex asymmetry is associated with head orientation preference (Rönnqvist, 1995; Rönnqvist et al., 1998). Both, in turn, are suggested to be related to vestibular lateralization. Newborns with low gestational age and/or low birthweight are found to exhibit a reduced head‐turning orientation compared to full‐term babies (Fox & Lewis, 1982; Gardner et al., 1977; Geerdink et al., 1994; Kurtzberg et al., 1979). Therefore, it is possible that PCBS such as prematurity might prevent the development of vestibular asymmetries in the last trimester, which in turn will disturb the development of other related behavioral lateralization (Previc, 1991, 1996). However, it should be noted that previous studies showed that the head orientation preference might only be partly related to the vestibular system, and other factors intrinsic to the developing fetus could be involved in the early postural asymmetries (Gardner et al., 1977; Geerdink et al., 1994). Postural and head asymmetries could also be a consequence of early gene expression (Fagard, 2013a, 2013b). Adopting the perspective that genetics is also a factor underlying the development of early asymmetries, PCBS may result in an increase of biological noise, which will reinforce the implication of chance in the development of lateralized behaviors such as the head orientation (Batheja & McManus, 1985). Nonetheless, independent of its origins, one can suggest that reduced head orientation among children with PCBS could lead, based on the cascade theory of handedness (Michel, 2021), to different trajectories of the development of handedness. Under a “development from” approach, where developmental traits emerge from an interaction between physiological processes and environmental experiences (Michel, 2021), weak postural asymmetries reduce the right‐hand use that is generally observed in most newborns, consequently reducing the sensorimotor, proprioceptive, and visual experience related to this hand during development. This could then lead to a higher probability of an atypical laterality during motor and language development.

**FIGURE 6:**
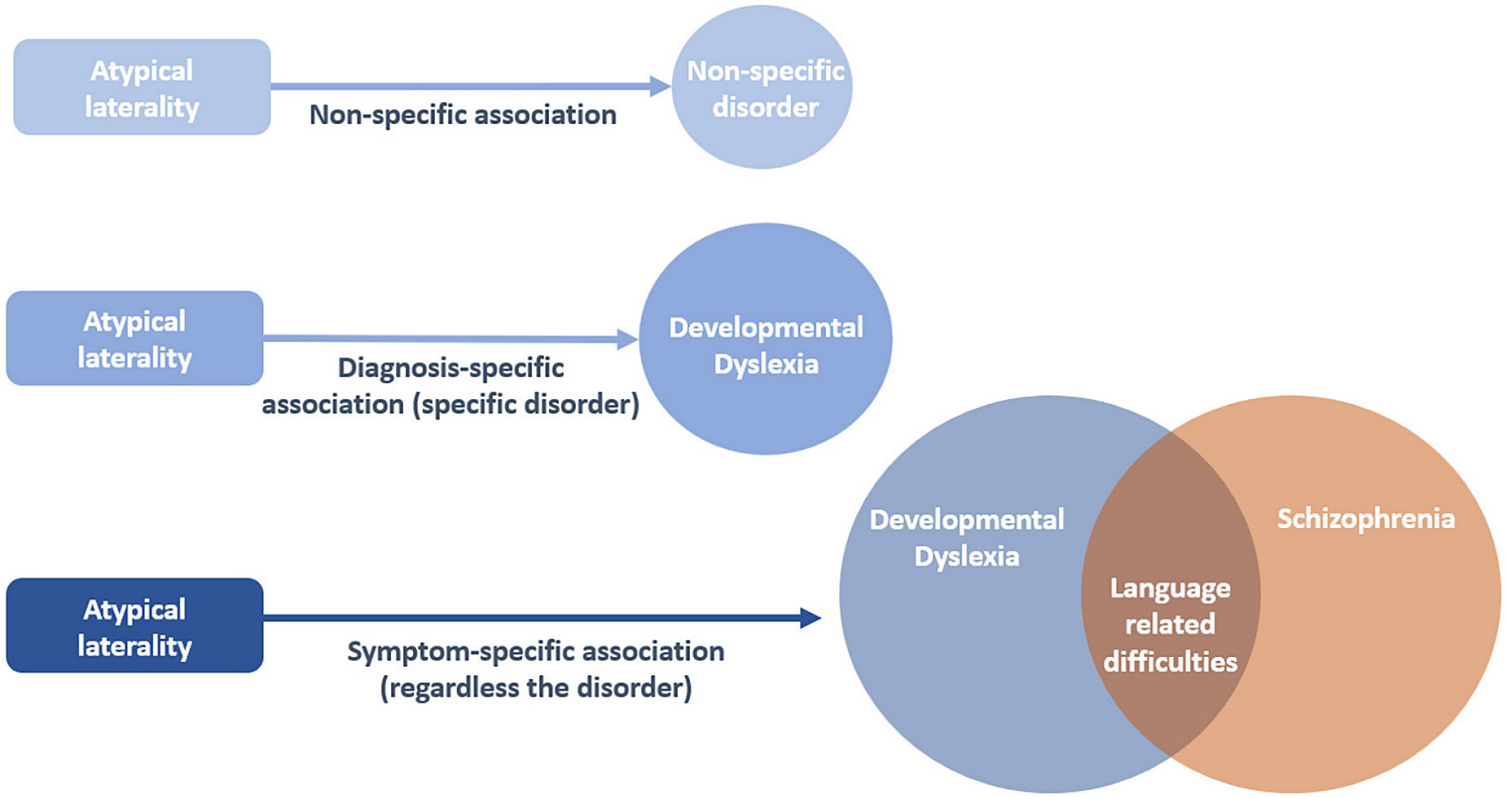
Example of the different possible associations between atypical laterality and disorders, suggested by Mundorf et al. (2021).

A final question remains concerning the nature of the association between atypical laterality and neurodevelopmental disorders. Mundorf et al. (2021) postulated on a theoretical level three different types of association between atypical laterality and disorders. The first is where several factors, such as genetics, increase the general risk of developing atypical asymmetries and non‐specific disorders (i.e., non‐specific association). The second is where it is postulated that specific factors increase the risk of atypical asymmetries related to a specific disorder, such as neural language networks in individuals with DD (i.e., diagnosis‐specific association). The third, which falls under a transdiagnostic perspective, proposes that specific factors are implicated in the development of atypical structural asymmetries and specific symptoms, independently of the diagnosis (i.e., symptom‐specific association; see Figure 6). Mundorf et al. (2021) suggested that the association between atypical laterality and disorders is more likely to be symptom‐specific rather than diagnosis‐specific (see also Ocklenburg & Mundorf, 2023). Evidence supporting the third type of association can be observed in two distinct disorders, DD and schizophrenia. Alterations in structural asymmetries among patients with schizophrenia who exhibit auditory verbal hallucinations are different from those in patients with no auditory hallucinations but similar to those found in individuals with other disorders such as DD (Mundorf et al., 2021). Furthermore, on a functional level, it has been found that children with severe symptoms of DD do not show a right ear advantage for language processing on a dichotic listening task unlike children with less severe symptoms (Helland et al., 2008). These findings suggest that a better approach to understanding the relation between atypical laterality, neurodevelopmental, and psychiatric disorders is a symptom‐specific approach, where the severity of the symptoms should be taken into consideration (Mundorf & Ocklenburg, 2021, p. 111; Mundorf et al., 2021).

Investigations into the nature of the association between perinatal adversities, atypical laterality, and neurodevelopmental disorders could benefit from a symptom‐specific approach. This approach would allow for a focused examination of the specific symptoms that might be impacted by PCBS and could be linked to both atypical laterality and neurodevelopmental disorders. Insight into these associations could be obtained from studies exploring the relationships between perinatal adversities, atypical handedness, and different neurodevelopmental disorders and their subtypes, such as language‐related disorders (e.g., DD with or without speech impairment) and motor‐related disorders (e.g., DCD).

Studies have shown that low gestational age and low birthweight are associated with atypical handedness (i.e., non‐right handedness) and DCD, but not with DD (Hamaoui, 2022, p. 150; Hamaoui et al., 2022; see also Bos & Tijms, 2012; Zwicker et al., 2013). Following a symptom‐specific approach, these findings imply a relationship between perinatal adversities, atypical laterality, and impairments related to motor skills, rather than those related to language.

In an attempt to address the association between PCBS and DCD but not DD, some authors speculated that the language impairments commonly observed in children with low gestational age and/or low birthweight may not reflect DD, but rather might be a consequence of more diffuse neurodevelopmental disorders (Bos & Tijms, 2012). DCD could be considered a candidate disorder since it is associated with learning disability and deficits in speech, language, arithmetic, attention, postural control, motor, and visuospatial skills (Wocadlo & Rieger, 2008). For instance, very preterm children with motor impairments show poorer literacy and numeracy performances than those without motor impairments (Wocadlo & Rieger, 2008). The relationship between motor and language development among children with PCBS can be looked at through an embodied cognition perspective. A disruption of cognitive development in children may not be related directly to PCBS but rather might reflect the consequence of motor impairments, where early sensorimotor interactions with the physical world are reduced (Oudgenoeg‐Paz et al., 2017). Therefore, PCBS could be more strongly related to motor rather than language development which would explain why they are more associated with DCD than DD.

Secondly, the association between atypical handedness and DCD but not DD may be explained by the hypothesis that handedness is more strongly linked to motor development than to language development. Michel, Babik, et al. (2013) proposed that language programming and speech processing are influenced by manual programming, where early sensorimotor experiences associated with hand preference shape later language abilities (based on the embodied cognition theory). They suggested that the link between handedness and language might be most observable among infants and young children, where hand performance measures are primarily employed. Supporting this assumption are longitudinal studies that have demonstrated that early handedness trajectories from 18 to 24 months predict later language abilities in young children at 5 years old (Gonzalez et al., 2020). Additionally, recent research indicates that functional lateralization varies across different aspects of cognitive functions like language, which is known to be multidimensional (Parker et al., 2022). For instance, functional language lateralization differs depending on specific language tasks, such as phonological decision, sentence generation, and syntactic decision tasks (Woodhead et al., 2019). Therefore, if a relationship between handedness and language exists, it would most likely be observed at the level of speech‐sound production (Michel, Babik, et al., 2013). This hypothesis could be supported by Becker et al. (2022), who found that handedness in gestural communication is associated with the asymmetry of the inferior arcuate sulcus, a homolog of Broca's area, in baboons.

Interestingly, while it is not consistent in the literature, a higher prevalence of atypical handedness was observed among individuals with language‐impaired disorders associated with articulation problems and motor impairments than those without motor‐related difficulties (Bishop, 1990b). Furthermore, there is a higher prevalence of left‐handedness among children with DCD than those with DD (Darvik et al., 2018). These findings present an opportunity to reconsider a hypothesis proposed by Bishop (1990a, p. 138–139) in which it is the motor functioning, rather than language, which may be linked with handedness. Results that appear to contradict this assumption are contained within the recent meta‐analyses conducted by Abbondanza et al. (2023) and Packheiser et al. (2023). They found a higher prevalence of non‐right‐handedness among individuals with reading and/or language impairment, and DD, compared to the control group (OR = 1.21 and OR = 1.57, respectively). Nonetheless, this finding may reflect the association between atypical handedness and motor instead of language impairments. Indeed, due to the fact that the authors had large samples consisting of 2503 and 4660 individuals with reading and/or language impairment, and DD, respectively, and since between 40% and 57% (depending on the severity) of individuals with DD present motor impairments (Chaix et al., 2007), this could explain why a significant association between atypical handedness and DD was found in these meta‐analyses. Altogether, it is possible that hand preference is dependent on the maturation of skilled motor functioning, hence the atypical handedness observed among children with motor immaturity (Bishop, 1990a, p. 139), wherein motor immaturity could be a consequence of PCBS, such as prematurity (Wallois et al., 2020).

Thus, it is plausible to propose that the association between perinatal adversities, atypical laterality, and neurodevelopmental disorders is more likely to be symptom‐specific, tied to motor impairments present across various disorders, rather than being associated with a specific diagnosis such as DCD or DD. Therefore, it could be suggested that atypical laterality is more likely to be observed among individuals with DD associated with visuospatial and motor impairment. This hypothesis can be further extended to include psychiatric disorders such as schizophrenia, where an association with atypical laterality has also been found (Berretz, Wolf, et al., 2020; Hirnstein & Hugdahl, 2014). From a developmental perspective, it is suggested that early motor impairments, which disrupt sensorimotor experiences, could contribute to an increased risk of developing schizophrenia spectrum disorders and related psychotic conditions (Poletti et al., 2019).

## 4 PERSPECTIVES

The theoretical framework presented in this article, which aimed to clarify the development and mechanisms underlying the association of atypical laterality and neurodevelopmental disorders, can pave the way for several future perspectives.

Firstly, preterm birth is usually considered as a single category on a clinical and research level (Frey & Klebanoff, 2016). However, prematurity is multifactorial and could be a consequence of genetics and/or environmental factors (Wadon et al., 2020). Furthermore, several risk factors leading to prematurity are well identified, which are related to maternal characteristics, reproductive history, and pregnancy characteristics (Frey & Klebanoff, 2016). Thus, differentiating between preterm births according to their etiologies becomes essential. Some authors suggested that new classifications of prematurity are required to replace the classical dichotomous one, that is, spontaneous or indicated (Frey & Klebanoff, 2016). The relationship between prematurity and atypical laterality prompts an important inquiry, which is whether prematurity, independent of its cause, is directly responsible for atypical laterality or whether specific factors contribute to both prematurity and atypical laterality. In a future study, it would be interesting to separate preterm children into different categories according to the causes that led to early birth. If there is a difference between these categories, it might indicate the potential mechanisms underlying their association with atypical laterality.

Secondly, and in the same vein, low gestational age and low birthweight led to different results when compared to poor neonatal health reflected by very low Apgar scores (Hamaoui, 2022, p. 147; Hamaoui et al., 2022). The former factors were associated with a higher prevalence of left‐hand preference and DCD, whereas the latter was related to hand preference and hand performance but not DCD. The association between low gestational age and low birthweight and DCD, in contrast to poor neonatal health assessed by the Apgar test, is supported by previous studies (Zwicker et al., 2013). These various PCBS reflect different etiologies and are not necessarily related (Behnke et al., 1987; Tiemeier & McCormick, 2019; Wainstock & Sheiner, 2022). One finding may provide insight into the underlying differences between these PCBS. Low gestational age and low birthweight and very poor neonatal health were significantly associated with hand preference, but only the latter tended to also be associated with hand performance. Handedness is multidimensional, where hand preference and hand performance are weakly correlated with each other (Castillo et al., 2020). These dimensions represent two distinct phenotypes influenced by different factors. On the one hand, they could be influenced by distinct biochemical pathways and genetic factors (Schmitz et al., 2019), while on the other hand, they are shaped by environmental factors related to asymmetric motor experiences and practice during early development (Marcori et al., 2019; Michel, 2021). Low gestational age and low birth weight may affect mechanisms that are solely implicated in the development of hand preference, whereas poor neonatal health may reflect other mechanisms related to both hand preference and hand performance. While some distinctions could be made between these PCBS events depending on their outcomes, it is difficult to draw strong conclusions from these results. The different outcomes obtained according to each of these PCBS may be due to the multifactorial origins of PCBS, which could be genetic, medical, environmental, and socioeconomic factors (Di Renzo et al., 2018; Wadon et al., 2020; Watterberg et al., 2015). Furthermore, the Apgar score is a composite measure of birth stress events and is a substitute for a range of isolated factors (Dragović et al., 2013), which naturally increases the difficulty of identifying precisely what it is being measured (Tiemeier & McCormick, 2019). Thus, it is difficult to try to uncover the exact underlying mechanisms that associate each of the PCBS with their outcomes. Nonetheless, these findings show the importance of testing each PCBS individually and raise the question of the classification of PCBS and their respective consequences.

Thirdly, even if an association between the Apgar score and atypical handedness could be found (Dragović et al., 2013; Hamaoui, 2022, p. 148–149; Schwartz, 1988), it is challenging to infer what the Apgar test measures precisely (Tiemeier & McCormick, 2019). To complement the Apgar test, administering additional neonatal assessments could provide more detailed information on newborn deficits. The Neonatal Behavioral Assessment Scale (NBAS; Brazelton & Nugent, 1995) could be appropriate as it aims to comprehensively assess the neonatal function over a full range of behaviors such as habituation, orientation, motoric processes, reflexes, and physiological response to stress (Costa et al., 2010). Another option, either as an alternative to or in conjunction with the NBAS, is the Hammersmith Infant Neurological Examination (HINE). The HINE is designed for infants aged 2–24 months (corrected age) and includes 26 standardized items that evaluate cranial nerves, posture, movement quality, quantity, tone, and reflexes (Romeo et al., 2016). The HINE provides valuable prognostic information on motor impairment and cerebral palsy, as well as an asymmetry score (Romeo et al., 2024) that is sensitive to detecting hemiplegia and may be useful for studying typical asymmetrical development.

Finally, since PCBS increases the risk of brain damage and cerebral palsy (Goldenberg & Culhane, 2007; Iliodromiti et al., 2014), it is important to take this variable into consideration. Controlling for this variable will allow for the testing of the previous theoretical frameworks (i.e., alteration of HPA axis and atypical vestibular lateralization) without having a confounding variable that could bias the results (i.e., early brain insult).

Another perspective is that to test the implication of the vestibular system in the development of behavioral and functional lateralization, future studies should target a direct measure of vestibular asymmetries, such as caloric irrigation combined with brain imaging (Dieterich et al., 2003; Janzen et al., 2008). Assessing vestibular lateralization could be done on children with PCBS and on children with neurodevelopmental disorders. Such studies would allow us to observe if a higher prevalence of atypical vestibular lateralization is present among children with PCBS, and if it is related to other atypical functional asymmetries generally found in individuals with neurodevelopmental disorders.

Additionally, handedness measurements should be treated as a continuum, not only dichotomous, and hand performance (e.g., strength of handedness) should be also considered (Bishop, 1984).

Lastly, it is important for future studies interested in investigating the relationship between laterality, neurodevelopmental, and psychiatric disorders to identify their subtypes. As aforementioned, it is most likely that atypical laterality is associated with specific symptoms (e.g., motor and visuospatial impairments) rather than with a diagnosis (e.g., DD, DCD, and schizophrenia).

## 5 CONCLUSION

Laterality is a consequence of an interaction between genetics and environmental factors that affect complex developmental cascades (Michel, 2021; Schaafsma et al., 2009), and some of these factors are shared with neurodevelopmental and psychiatric disorders (Berretz, Wolf, et al., 2020). In critical periods during development, perinatal adversities could disrupt lateralization through epigenetic and developmental mechanisms (Kwon et al., 2015; Michel, 2021). Under this perspective, perinatal adversities (e.g., maternal stress and birth complications) might induce epigenetic modifications, which will affect in turn the ontogenesis of brain asymmetries, including handedness (Schmitz et al., 2017). It was suggested that these events could be potential candidates for explaining the higher prevalence of atypical laterality in neurodevelopmental disorders. The mechanisms underlying this association remain to be clarified but are likely either stress (i.e., via the alteration of the HPA axis) and/or disrupted vestibular lateralization. Undoubtedly, future studies will be conducted to better understand all of these associations.

## AUTHOR CONTRIBUTIONS

**Jad Hamaoui:** Conceptualization; project administration; visualization; writing – original draft; writing – review and editing. **Sebastian Ocklenburg:** Supervision; visualization; writing – review and editing. **Hervé Segond:** Funding acquisition; supervision; visualization; writing – review and editing.

## FUNDING INFORMATION

The Grand‐Est Region (Alsace, France) financially supported the present work [18P07546—18_GE4_066—LATERALCOG].

## CONFLICT OF INTEREST STATEMENT

No potential conflict of interest was reported by the author(s).

## Data Availability

Data sharing is not applicable to this article as no data sets were generated or analyzed during the current study.
